# Breaking the bind: PfEMP1-specific antibodies in cerebral malaria

**DOI:** 10.3389/fimmu.2025.1681852

**Published:** 2025-10-30

**Authors:** Josephine P. Banda, Isobel S. Walker, Tonney Nyirenda, Elizabeth H. Aitken, Stephen J. Rogerson

**Affiliations:** ^1^ Department of Pathology, Kamuzu University of Health Sciences, Blantyre, Malawi; ^2^ Molecular Core Laboratory, Blantyre Malaria Project, Blantyre, Malawi; ^3^ Department of Infectious Diseases, The Peter Doherty Institute for Infection and Immunity, The University of Melbourne, Melbourne, VIC, Australia; ^4^ Department of Microbiology and Immunology, The Peter Doherty Institute for Infection and Immunity, Melbourne, VIC, Australia

**Keywords:** *Plasmodium falciparum* Erythrocyte Membrane Protein 1 (PfEMP1), cerebral malaria (CM), severe malaria (SM), antibodies, naturally acquired immunity

## Abstract

Antibodies against *Plasmodium falciparum* erythrocyte membrane protein 1 (PfEMP1) on infected erythrocytes (IEs) play a central role in naturally acquired protection against cerebral malaria (CM), yet the determinants of effective humoral immunity remain incompletely defined. We review evidence from seroepidemiological, functional, and mechanistic studies demonstrating that antibodies to endothelial protein C receptor (EPCR)‐binding cysteine-rich interdomain regions (CIDR)α1 and Duffy binding-like (DBL)β domains associated with dual EPCR and intercellular adhesion molecule 1 (ICAM1) binding correlate with reduced risk of CM, while responses to rosetting‐associated domains (DBLα, CIDRγ) and other domains are less well characterized. We synthesize findings on antibody kinetics—early, durable responses to Group A variants versus delayed, transient responses to Groups B and C—and on effector mechanisms including opsonic phagocytosis, complement activation, and Fc glycosylation. We highlight methodological challenges in measuring PfEMP1‐specific immunity, such as antigenic switching, differences between assays using single domains and native protein on IEs, and the need for physiologically relevant 3D vascular models. Finally, we identify key research priorities: mapping immunodominant epitopes across variant repertoires; longitudinal cohort studies to track antibody maturation and post‐translational modifications; and the development of broadly inhibitory monoclonal antibodies. Addressing these gaps will be critical for designing vaccines and therapeutics that harness protective antibody functions to prevent CM.

## 1 Introduction

Malaria remains a significant global health challenge, with over 200 million cases annually, disproportionately affecting resource-constrained regions, mostly in sub-Saharan Africa (1). Vulnerable populations, including children under 5 and pregnant women, bear the greatest burden, with severe consequences like high mortality rates, neurological complications, and adverse pregnancy outcomes. Malaria presents across a broad clinical spectrum, ranging from asymptomatic parasite carriage to uncomplicated malaria (UM), and progressing to severe, life-threatening disease. Severe malaria (SM) includes complications such as severe malarial anemia (SMA) and neurological syndromes like cerebral malaria (CM). CM, the most severe form of *Plasmodium falciparum (P. falciparum)* malaria, is clinically defined as unarousable coma not attributable to other causes in the presence of parasitemia (1).

*P. falciparum*, the most virulent species of malaria parasites infecting humans, is responsible for most of the severe disease and mortality (1, 2). A key contributor to the virulence of *P. falciparum* is the binding of infected erythrocytes (IEs) to the vascular endothelium, causing sequestration of the parasite in the microvasculature of various tissues. By preventing splenic clearance, sequestration aids parasite survival. Sequestration of IEs in the brain’s microvasculature is a defining feature of CM pathogenesis (3).

**Figure 1:**
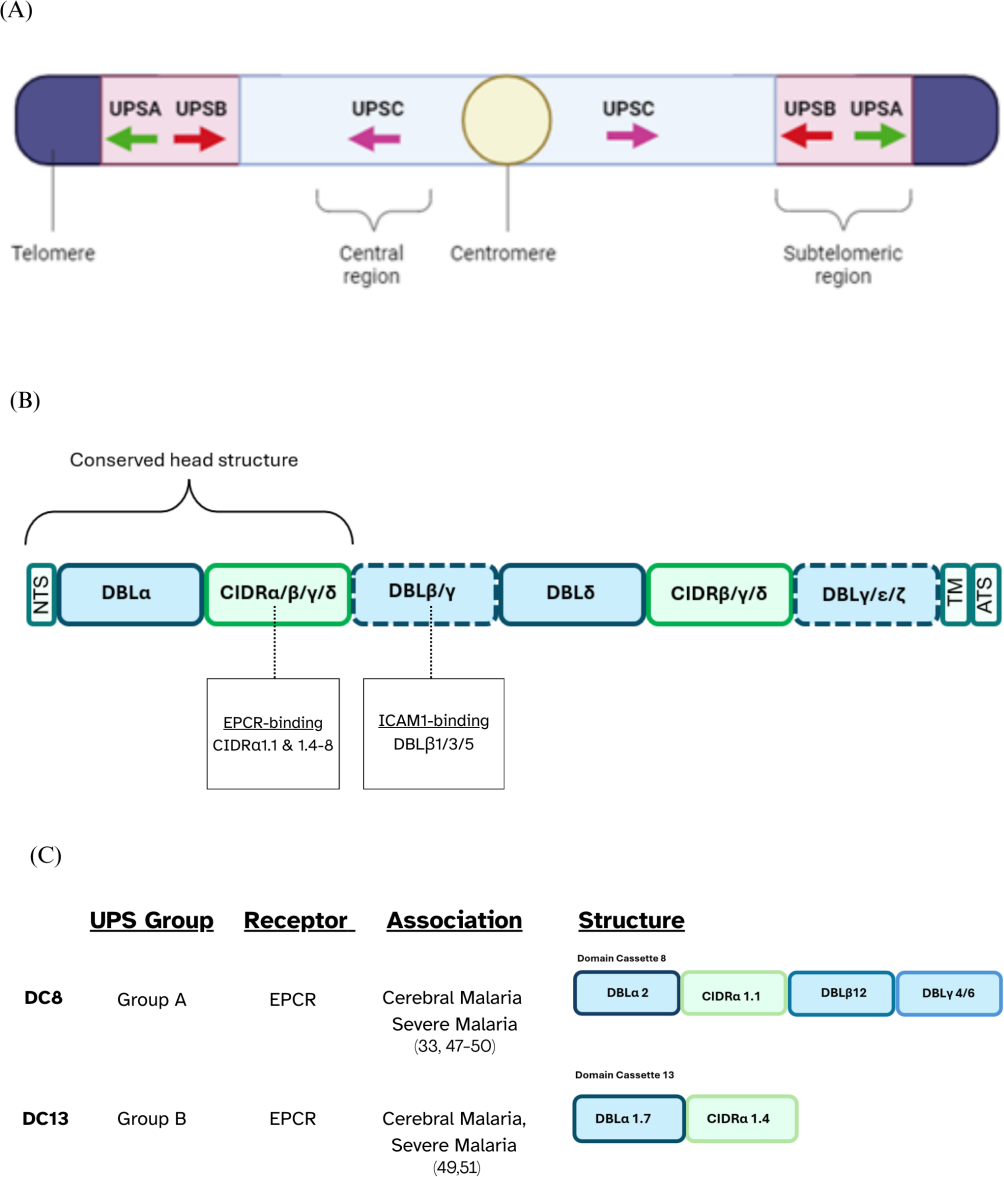
PfEMP1 classification overview. **(A)** UPS classification illustrating the chromosomal locations of *var* genes along with the direction of their transcription, depicted using arrows. **(B)** Schematic representation of the typical PfEMP1 structure, with dotted lines indicating domains that may or may not be present depending on the specific variant. **(C)** Schematic representation of domain cassettes 8 and 13 and their UPS group and binding phenotype.

Sequestration is mediated by *Plasmodium falciparum* Erythrocyte Membrane Protein 1 (PfEMP1), a species-specific, highly polymorphic protein that is predominantly expressed on the surface of IEs during the blood stage of infection (4, 5). PfEMP1 proteins are encoded by approximately 60 *var* genes per genome that undergo frequent recombination to enhance their antigenic diversity and immune evasion capabilities (4, 6). Though diverse, the *var* genes that encode most PfEMP1s can be grouped based on upstream promoter sequences (UPS), chromosomal location, and transcription direction (**Figure 1A**) into 3 main groups: Group A, B, C, and 2 intermediary groups: Group B/A and UPS B/C.

The extracellular ectodomain of PfEMP1 has a modular structure, primarily composed of 2–10 tandemly arranged protein domains named the Duffy Binding-Like domains (DBL) and the Cysteine-Rich Interdomain Regions (CIDR). PfEMP1’s antigenic diversity is driven by the variation in the number, arrangement and sequences of the DBL and CIDR domains present in the ectodomain of different PfEMP1s (4, 7). Sequence similarities allow for the classification of DBL domains into distinct classes, including α, β, γ, δ, ϵ, and ζ, while CIDR domains are grouped into classes such as α, β, γ, δ, and pam. Each of these major classes is further subdivided into subclasses denoted by numbers, e.g., DBLα1 (5). Over 95% of PfEMP1s feature a head structure composed of tandem DBLα and CIDR domains adjacent to the N-terminal segment (NTS) (5). The central region of PfEMP1 often contains multiple, alternating DBL and CIDR domains, followed by a transmembrane region and an acidic-tail segment at the C-terminus (4, 5, 8) (**Figure 1B**).

There are specific combinations of domains which are seen in different PfEMP1s. Domain Cassettes (DCs) are defined as structural alignment of two or more adjacent DBL and CIDR domains within PfEMP1 proteins that frequently occur together in at least three *P. falciparum* genomes (5). Some DCs are known to bind to specific endothelial receptors, and it is possible that these conserved arrangements have evolved to facilitate a survival advantage for the parasite. For example, DC8 which consists of DBLα2 – CIDRα1.1 – DBLβ12 – DBLγ4/6 binds to Endothelial Protein C Receptor (EPCR) via a conserved CIDRα1.1 domain (**Figure 1C**).

The clinical manifestations of *P. falciparum* infection are influenced by the parasite’s ability to bind specific host receptors, which differ in abundance and distribution across tissues. While many of these receptors are found on the vascular endothelium, others involved in rosetting or placental malaria are located on uninfected erythrocytes and placental syncytiotrophoblasts, respectively. For instance, intercellular adhesion molecule 1 (ICAM1) is abundant in the brain, chondroitin sulfate A (CSA) is found on placental syncytiotrophoblasts, and Cluster of Differentiation 36 (CD36) is widely distributed throughout many tissues in the body (9–11). The ability of a particular PfEMP1 variant to bind to specific receptors determines where IEs sequester, driving organ-specific complications. This selective binding is critical to the manifestation of CM, in which PfEMP1 variants with high affinity for brain-expressed receptors promote sequestration in the microvasculature of the brain (4, 12, 13).

### 1.1 The pathogenesis of cerebral malaria

Despite standardized diagnostic criteria, distinguishing true CM from other causes of coma remains difficult in high-transmission settings, where incidental parasitemia is prevalent. The clinical definition of CM (Blantyre coma score ≤2, parasitemia, and exclusion of other causes (1)) misclassifies approximately a quarter of cases, which have alternative causes like meningitis, highlighting the prevalence of incidental parasitemia (3). Pathophysiologically, CM can be categorized into 3 subtypes: sequestration only, sequestration with microvascular pathology, and no sequestration—the latter likely representing non-malarial causes of comas (3, 14). Retinal findings, such as hemorrhages, vascular whitening, and other vascular changes, are strongly associated with sequestration in true CM, while the absence of these points to non-malarial causes of coma (3). These features make fundoscopic examination a practical, non-invasive tool to confirm CM (15, 16).

**Figure 2:**
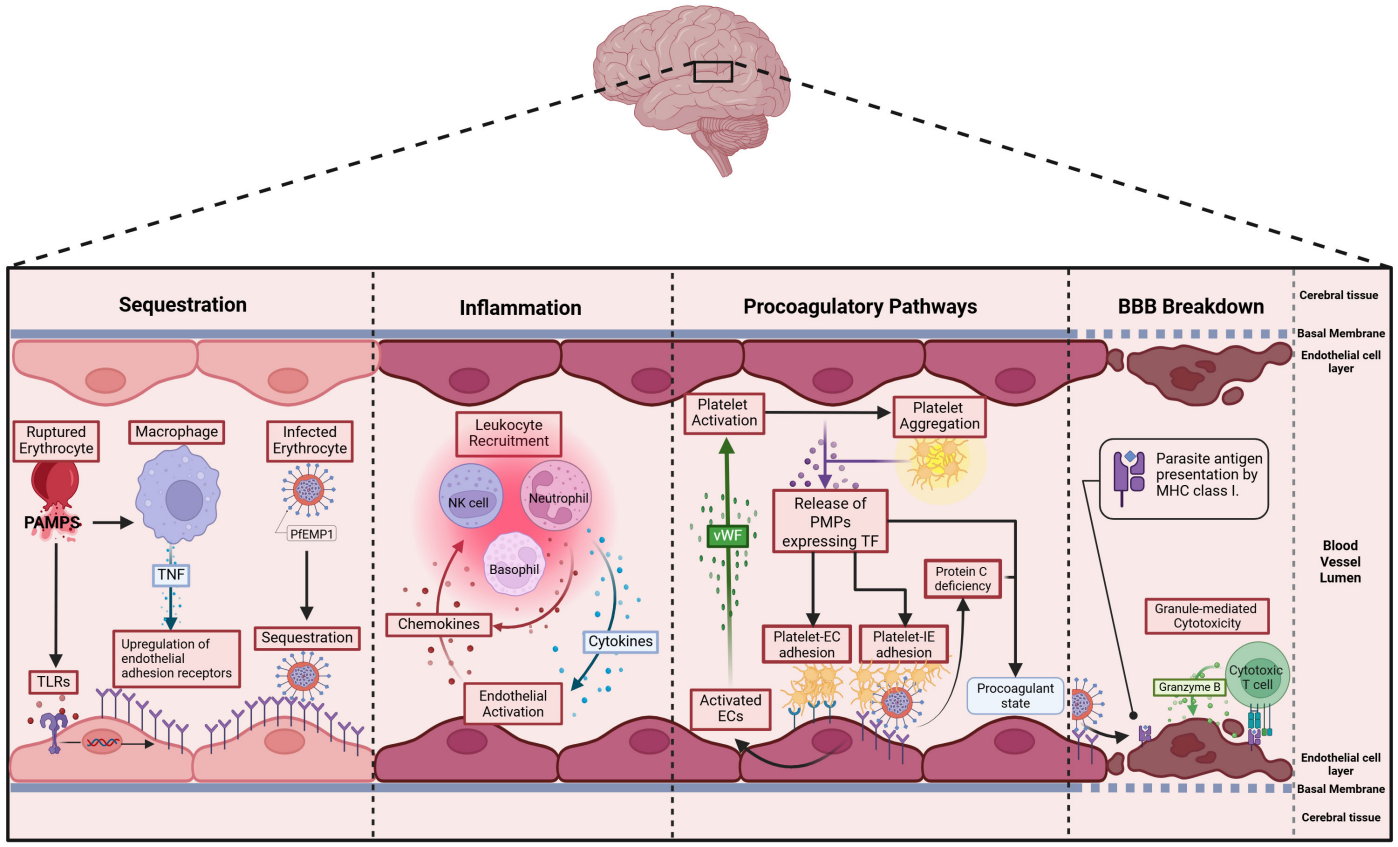
Postulated pathophysiology of cerebral malaria: 1. Sequestration – *P. falciparum* pathogen-associated molecular patterns (PAMPs) activate Toll-like receptors (TLRs) on endothelial cells and macrophages, upregulating endothelial adhesion molecules. TNF from macrophages amplifies this, promoting PfEMP1-mediated IE binding and further endothelial activation in the brain (17, 18). 2. Inflammation – Activated endothelial cells (ECs) secrete chemokines that recruit leukocytes, which in turn release inflammatory cytokines like TNF, further promoting sequestration. This cycle amplifies inflammation as recruited immune cells continue to produce cytokines and chemokines, sustaining the response (19). 3. Procoagulatory pathways – Activated ECs release von Willebrand Factor (vWF), which binds to glycoproteins on platelets, triggering their activation (20). Activated platelets aggregate and express various surface receptors—including tissue factor—that enhance adhesion to both ECs and IEs, forming multimeric complexes (21). Additionally, sequestration reduces the abundance and activation of protein C (22). 4. Blood-Brain Barrier (BBB) Breakdown - Cytotoxic T cells infiltrate the brain’s microvasculature, recognize antigens on endothelial cells via MHC-I (23), and release granzyme B to trigger apoptosis (24). This cytotoxic activity disrupts the blood-brain barrier, contributing to vasogenic oedema and neurological damage, and may result in brain swelling (25).

In CM, pathology is partly driven by blood-brain barrier breakdown, a process involving multiple interrelated mechanisms. Sequestration of IEs, inflammation from inflammatory cytokines, endothelial activation, and dysregulated coagulation leading to microvascular thrombosis can all contribute to tight junction disruption (**Figure 2**) [Reviewed in Jensen et al. (17)]. The expression of endothelial adhesion receptors is upregulated by two responses to pathogen associated molecular patterns (PAMPS) released during schizont rupture, namely the sensing of PAMPs by Toll-like receptors and TNF production by macrophages. Thus, the upregulation of endothelial adhesion receptors leads to further sequestration of IEs (18, 19). Sequestration activates endothelial cells, which in turn produce chemokines that recruit leukocytes. These leukocytes further amplify local inflammation by releasing additional chemokines (20). Cytotoxic T-cells are also recruited and can recognize endothelial-bound antigens via MHC-I sensing (21). They induce endothelial cell apoptosis through Granzyme B (22), compromising the integrity of the blood–brain barrier. During pathogenesis, a procoagulatory microenvironment develops due to two main factors: first, sequestration reduces the availability and activation of Protein C (23); second, activated endothelial cells release Von Willebrand factor, which activates platelets (24). These activated platelets then aggregate and bind to endothelial receptors or IEs (25). *In vitro* and *ex vivo* data suggests that IEs expressing ICAM1 and EPCR dual-binding PfEMP1 variants are internalized by brain endothelial cells via an ICAM1–dependent mechanism, leading to endothelial cell swelling and impaired BBB integrity (26). Additionally, focal hemorrhages further weaken the blood-brain barrier, causing plasma and protein leakage into brain tissue, which promotes cerebral swelling, a severe and potentially fatal complication of CM (27, 28). Sahu et al. (29) showed that increased parasite biomass (as indicated by higher PfHRP2 levels) and the elevated expression of EPCR-binding PfEMP1 variants are key determinants driving brain swelling in CM.

## 2 Structural diversity of PfEMP1s associated with cerebral malaria and their host receptor interactions

### 2.1 Sequestration

Sequestration in the cerebral microvasculature is a hallmark feature of CM. There isn’t a single PfEMP1 variant exclusively linked to the development of CM. Instead, multiple PfEMP1 variants are differentially transcribed in SM and CM (30–32). Given the high diversity of PfEMP1, research has focused on the binding phenotypes of different PfEMP1. Among these, EPCR and ICAM1-binding variants can bind brain endothelial cells *in vitro* and often associated with CM, making them the focus of the following discussion. However, they are not always expressed in CM and may not be the only PfEMP1 variants involved in its pathology.

Many studies have observed an association between infections caused by *P. falciparum* parasites that express *var* genes encoding PfEMP1 proteins from Group A and B/A and SM including CM (26, 30, 33–36). The receptor-binding phenotypes of some Group A and B/A PfEMP1 proteins can be directly linked to the pathogenesis of CM. Some PfEMP1 variants are capable of simultaneously binding to both EPCR and ICAM1 (37, 38) and CM is associated with *P. falciparum* expressing dual EPCR- and ICAM1-binding PfEMP1 variants, as shown by both upregulation of the corresponding *var* genes (39, 40) and IEs which bind microvascular endothelium (38, 41). Notably, these “dual-binding” proteins are primarily found in Group A and Group B/A (38).

EPCR is a receptor located on vascular endothelial cells, and PfEMP1 variants with CIDRα1 domains can bind EPCR, a feature associated with SM and CM (33, 40, 42). Phylogenetically, CIDRα1 sequences are grouped into subclasses (CIDRα1.1–1.8), of which CIDRα1.1 and 1.4–1.8 bind EPCR, while CIDRα1.2 and 1.3 do not and may occur only in pseudogenes.

DBLβ1/3/5 domains are known to bind ICAM1 (38, 43–45). In dual-binding PfEMP1 variants, ICAM1-binding DBLβ domains are downstream of EPCR-binding CIDRα1 domains (38). The “DBLβ motif”, a short amino acid sequence within DBLβ domains of specific PfEMP1 proteins, is associated with a dual binding PfEMP1 to EPCR and ICAM1. This motif is present in Group A and some Group B/A variants and is transcribed by isolates from children with CM (26, 46). Although transcript levels of *var* genes that encode both Group A and Group B PfEMP1 were elevated in CM cases (41), it remains unclear what fraction of these transcripts correspond to EPCR–ICAM1 dual binders versus ICAM1–only binders. In other words, it is not yet known whether ICAM1–binding PfEMP1 that lack EPCR binding are associated with disease. To address this, future studies should directly compare *var* gene transcription and surface expression of ICAM1–only versus dual-binding PfEMP1 in cerebral and non-cerebral cases, and evaluate these isolates' adhesive capacity under physiological flow conditions.

DC13 is an EPCR-binding domain cassette found in certain Group A PfEMP1 proteins, which is composed of DBLα1.7 and CIDRα1.4 (**Figure 1C**) (9, 43). High transcription of *var* genes encoding DC13 is observed in both African children and Indian adults with SM, including CM, suggesting that this association is not geographically restricted (47–50). Additionally, DC13-encoding *var* genes show higher transcription levels in CM compared to SMA, reinforcing their stronger link to CM (33, 48, 50–52). DC8 is an EPCR-binding PfEMP1 variant, primarily found in Group B PfEMP1 proteins, consisting of four domains: DBLα2, CIDRα1.1, DBLβ12, and DBLγ4/6 (4). Although DC8 transcription is elevated in CM it is also upregulated in individuals with broader SM manifestations (49, 51).

In CM, the transcription of DBLα from Group A *var* genes is upregulated compared to UM (30, 36). This includes DBLα1.1, which is typically adjacent to a CIDRα1.4/6/7 domain that facilitates binding to EPCR (4, 53) and DBLα1.6/8 domains that typically feature in rosetting types, which will be discussed in the following section (4, 53).

Platelet and endothelial cell adhesion molecule 1 (PECAM-1) binds PfEMP1 on infected erythrocytes. *In vitro* studies demonstrate adhesion to recombinant and transfected PECAM-1, and many field isolates, including those from children with severe malaria, show measurable but generally low binding (54). PECAM-1 is also expressed in the microvasculature of the human brain (55), providing a biologically plausible site for such interactions. Genetic association studies have further linked PECAM-1 polymorphisms with susceptibility to cerebral malaria, supporting its potential relevance to severe disease (56, 57). However, unlike EPCR, direct evidence from human autopsy material demonstrating colocalization of sequestered parasites with PECAM-1 in brain microvessels is lacking (23). This gap highlights PECAM-1 as a plausible but unconfirmed contributor to cerebral sequestration, and an important target for future exploration.

### 2.2 Rosetting

PfEMP1 not only mediates sequestration but can bind IEs to uninfected erythrocytes, forming clusters in a process known as rosetting. Isolates from individuals with CM have been shown to form rosettes *in vitro* (58–60). However, the contribution of rosetting to pathogenesis remains unclear due to conflicting findings, which may reflect geographical differences. In sub-Saharan African cohorts, significantly higher rosetting rates were observed in CM compared to UM (58, 61), and rosetting was elevated in SM compared to UM, regardless of syndrome subtype (59, 60). In contrast, a study from Papua New Guinea found similar rosetting rates in CM and UM (62), while a Thai study reported the highest rosetting frequencies in CM compared to both UM and non-cerebral SM (63).

The mechanisms by which rosetting might contribute to malaria pathogenesis remain unclear. One hypothesis is that rosettes impede phagocytosis of IEs. The larger size and complex structure of rosettes can physically hinder phagocytes from engulfing infected cells (64, 65). When rosetting is disrupted, parasites become more susceptible to phagocytosis, but intact rosettes require multiple phagocytes for clearance, potentially leading to phagocyte exhaustion in hyperparasitemic malaria patients (66).

Assessing the exact contribution of rosettes to disease severity is challenging, as they are difficult to observe in autopsy samples, making it hard to confirm their presence and impact in affected organs. Furthermore, rosetting involves multiple host factors, such as ABO blood group and complement components (67–69), which vary between individuals and populations. There may not be a universal mechanism linking rosetting to severe disease, as both host and parasite factors contribute to its heterogeneity. These limitations underscore the need for innovative approaches, such as 3D microvessels with precise control over vessel architecture and blood flow (70), to better replicate *in vivo* rosette dynamics.

Most rosetting variants are encoded by Group A *var* genes and typically feature domain combinations—such as DBLα1.5/6/8 paired with CIDRβ/γ/δ—that form the rosetting-associated head structure (4, 71–75). Given that transcription of these Group A *var* genes is upregulated in CM (26, 33, 35), this supports the idea that rosetting may contribute to pathogenesis. However, several knowledge gaps remain. First, not all *var* genes that give rise to the rosetting phenotype have been definitively identified, and it is unclear whether currently known associations capture the full diversity of rosetting PfEMP1 variants. Second, transcriptomic data provide only partial insight into protein expression; mRNA levels don’t always translate to surface-expressed PfEMP1, and the relationship between *var* gene transcription and PfEMP1 display remains incompletely understood. Third, parasites sampled from peripheral blood may not reflect the phenotype of sequestered IEs in critical organs, where rosetting is presumed to exert its pathological effects. The second and third challenges, although discussed in this section, represent broader obstacles to understanding all PfEMP1 and not only rosetting variants.

## 3 Antibodies to PfEMP1 in cerebral malaria

Antibodies targeting PfEMP1 are likely essential for malaria immunity as they could prevent the sequestration of IEs and facilitate IE clearance. PfEMP1 appears to be the immunodominant surface antigen on IEs, as suppression of PfEMP1 expression significantly reduced IgG binding by plasma from malaria-exposed individuals (76). Effective immunity may result from acquisition of antibodies to a broad range of PfEMP1 variants, or from the acquisition of strain-transcending antibodies against specific binding phenotypes (75, 77).

Individuals living in malaria-endemic regions develop naturally acquired immunity to *P. falciparum* through repeated infections, leading to the production of antibodies against key parasite antigens, including PfEMP1 (78, 79). This immunity is associated with reduced disease, with adults and older children developing a broader, more robust antibody response to PfEMP1 variants due to cumulative exposure (80, 81) The best evidence for the protective effect of PfEMP1 antibodies comes from placental malaria. Pregnant women develop antibody to the PfEMP1 VAR2CSA in a gravidity dependent manner, and development of these antibodies is associated with declining prevalence and density of placental malaria infection (Reviewed in Rogerson et al. (82)).

Studies indicate that PfEMP1-specific antibodies develop sequentially in response to different parasite variants. The earliest acquired antibodies target Group A PfEMP1 variants, which are associated with SM, followed by Group B and C variants, which are linked to UM (78, 79, 83). For example, Tessema et al. (84) demonstrated that young Papuan New Guinean children, age 1–3 years old, mainly develop antibodies to Group A PfEMP1, indicating early infections involve these variants. With age and repeated exposure, their immunity broadens to include Group B/C PfEMP1s, potentially reflecting progressive immunity to SM (84).

Sequential acquisition of antibodies toward Group A, B and C variants is true for CIDRα domains that bind to EPCR and CD36. In malaria-endemic regions, antibodies to CIDRα1 are present at birth, due to maternal antibody transfer, but decline by around six months of age (85). Children then begin to acquire IgG antibodies against EPCR-binding CIDRα1 variants, such as CIDRα1.7 and CIDRα1.8, earlier than IgG targeting CD36-binding variants (86, 87). Transcription of CIDRα1.7 was particularly associated with brain swelling in Malawian children (40). CIDRα1.7 elicited the highest IgG antibody levels among all the CIDR domain variants tested in young children and a larger proportion of children in the cohort had detectable IgG to CIDRα1.7 compared to other variants (86). This early antibody acquisition may reflect a parasite fitness advantage from expression of EPCR‐binding PfEMP1 variants, which mediate microvascular adhesion (33, 42, 88). PfEMP1 variants that are both common in circulating parasites and initially unopposed by antibodies are favored in early infection. As children develop specific IgG (such as against CIDRα1.7) these antibodies target the most widely circulating variants, gradually eroding their fitness advantage (89, 90).

Young children are highly vulnerable to CM, likely because of the limited antigenic breadth and functional capacity of their antibodies, predisposing them to high parasite burdens (90). Travassos et al. (91) highlight syndrome-specific differences: children with CM exhibited more frequent and wider gaps in seroreactivity than those with SMA and UM.

### 3.1 Antibody responses to EPCR-binding PfEMP1

Children recovering from SM —including CM and SMA—exhibit increased PfEMP1 antibody levels during convalescence, particularly targeting EPCR-binding CIDRα1 domains (91, 92). These findings suggest that episodes of severe disease may drive the acquisition of immunity to EPCR-binding PfEMP1 variants (91). Notably, Nunes-Silva et al. (93) reported that children with CM failed to boost antibody responses against parasites expressing the EPCR-binding PfEMP1 VAR19 or recombinant proteins containing VAR19’s EPCR binding CIDRα1.1 domain following infection. While this appears to contrast with previous findings of post-infection increases in antibody to EPCR-binding PfEMP1 variants, the difference may reflect the use of a single EPCR-binding PfEMP1 in the study (93). The apparent lack of boosted immunity to that variant may simply reflect antigenic differences between the PfEMP1s circulating in Benin and the variant tested. However, while convalescent children with CM develop a boost in IgG to EPCR-binding PfEMP1 domains (91, 92), they remain at increased risk of subsequent SM episodes (94). This may reflect immunological gaps, as Travassos et al. (91) demonstrated that children with CM lack IgG breadth to certain PfEMP1 subsets, and Rambhatla et al. (92) showed that convalescent boosts can be non-broadly reactive or transient. The persistence of these antibody “blind spots” may underlie why even boosted responses do not necessarily translate into durable protection from recurrent SM.

Across diverse populations, antibodies to the EPCR-binding CIDRα1 domains of PfEMP1 are significantly higher in UM than in SM, including CM, suggesting that these antibodies may play a protective role (76, 95, 96).

Antibody specificity plays a critical role in mediating protection against severe disease. In one study of IgG levels to 32 PfEMP1 domains—selected based on their differential transcription in SM compared to UM— individuals with UM had significantly higher IgG against 15 of 22 SM-associated PfEMP1 domains compared to those with SM (95). Of the domains eliciting significantly higher IgG responses, CIDRα1.6 was one of the three PfEMP1 domains that most effectively distinguished uncomplicated from severe cases (95). This suggests that IgG to CIDRα1.6 may contribute to protection from SM. Another study, which focused specifically on EPCR-binding DC13 (DBLα1.7-CIDRα1.4) found no significant differences in IgG1 nor IgG3 responses to DC13 between CM and UM (96). However, these IgG1 and IgG3 levels to DC13 did significantly increase in the CM cohort from admission to convalescence (96). Similarly, Kessler et al. (97) assessed IgG seroreactivity to 61 3D7-derived PfEMP1 domains using a proteome microarray in children with retinopathy-positive CM or UM and found no differences in antibody responses to EPCR-binding DC8 domains between the groups (97). This suggests that not all antibodies to EPCR−binding domains confer equal protection and echoes the “gaps in seroreactivity” described by Travassos et al. (91).

Retinopathy in CM provides a non-invasive window into brain pathology, reflecting both microvascular sequestration and hemorrhagic events occurring in the cerebral microvasculature (3, 16). Additionally, Joste et al. (98) reported that among children with CM, those with retinopathy had significantly lower IgG responses to the EPCR-binding CIDRα1.4 than children without retinopathy, even though the expression of *var* genes encoding CIRDα1.4 binding domains and *in vitro* cytoadherence levels of isolated IEs to EPCR were similar between the groups (98). Although antibodies to EPCR-binding CIDRα1.4 domains did not differ overall between all CM cases compared to UM (98), their lower levels in CM children with retinopathy compared to those without suggest a potential role in modulating severity, rather than conferring outright protection from CM.

The CIDRα1.1 domain of DC8 is known to bind to EPCR, however, other domains of DC8 may also contribute to vascular adhesion, via additional receptors. Recombinant DC8 exhibited stronger binding to EPCR and to an immortalized human microvascular endothelial cell line compared to the CIDRα1.1 domain of DC8 alone (93). Notably, antibodies targeting the multi-domain DC8 protein fully blocked binding to recombinant EPCR but only partially inhibited endothelial cell adhesion (93). This implies that domains of DC8 other than CIDRα1.1 could interact with unknown receptors, highlighting alternative adhesion pathways contributing to IE binding in CM (93). This notion of alternative adhesion pathways is further supported by findings from a separate study, which reported that children with UM had significantly higher IgG reactivity to DBLα2 and DBLγ6 domains (both of which are part of DC8) compared to those with CM (76). In contrast, IgG responses to CIDRα1 (the EPCR-binding domain) and DBLβ12 did not differ between the groups (76). These findings suggest that antibodies targeting only CIDRα1.1 may not block all adhesion mechanisms *in vivo*, and that antibodies against adjacent domains such as DBLα2 and DBLγ6 may contribute to protection against CM by interfering with alternative binding interactions beyond EPCR. However, further research is required to confirm these hypotheses.

### 3.2 Antibody responses to ICAM1-binding PfEMP1

Acquisition of antibodies to ICAM1–binding PfEMP1 domains differs between Group A and Groups B and C. Olsen et al. (79) report that in healthy children from Ghana, Group A DBLβ domains, which are more conserved and often associated with CM pathogenesis, tend to elicit early and sustained antibody responses, as seen in longitudinal cohorts (79, 99). These responses are characterized by prompt seroconversion and evidence of long-lived antibody memory, while responses to Groups B and C develop later and increase gradually with repeated exposure (79).

The role of DBLβ domains, particularly those encoding ICAM1-binding regions, as targets of the immune response in CM is complex and multifaceted.

In a study using an ICAM1-binding DBLβ3 domain from a Group A PfEMP1 variant with an EPCR-binding CIDRα1 domain, higher IgG levels against the DBLβ3 at enrolment were significantly associated with a reduced risk of high‐density clinical malaria (fever + ≥10,000 parasites/µL) and of progression to SM during follow-up (69 weeks) (84). While this longitudinal evidence suggests that antibodies to this DBLβ3 may protect against malaria, it remains unclear whether this protection extends to CM. Future studies should stratify SM cases by clinical phenotype to determine if these antibodies confer similar protection against CM, considering the potential for distinct pathogenic mechanisms between CM and other SM manifestations.

While children with CM and UM of similar age show no significant differences in DBLβ-specific IgG titers (98, 100, 101), they do differ in antibody function. Two separate studies investigated phagocytic activity against ICAM1-binding PfEMP1. In Benin, where the cohort included children with SM including CM, and in Malawi, which focused exclusively on CM, individuals with UM showed greater phagocytic activity against DBLβ-coated beads and ICAM1-binding IEs, respectively (101, 102). Additionally, among the four recombinant ICAM1-binding domains assessed, antibodies from children with UM showed significantly higher inhibition of ICAM1 binding to a Group A DBLβ domain, compared to antibodies from children with SM or CM (101). These findings suggest that antibody-mediated inhibition of ICAM1-binding and enhanced opsonic phagocytosis may contribute to protection against SM (101). These functional differences may be influenced by antibody features including IgG subclass. IgG1 and IgG3 are effective in driving phagocytosis, but it is unknown how antibody subclass influences binding inhibition. IgG1 and IgG3 antibody titers against multiple ICAM1–binding DBLβ domains were associated with protection against severe disease in Beninese children (101). This suggests that individuals in Benin acquire functional antibodies that can bind to multiple DBLβ domains and may be protective.

### 3.3 Antibody responses that disrupt rosetting

Several studies have demonstrated that immune sera from malaria-exposed individuals can reverse rosetting *in vitro*, particularly in severe disease including CM (58, 67, 73). One study found that IgG to the SM-associated DBLα1.5 domain (selected based on its higher transcription in SM compared to UM) was significantly higher in UM compared to SM (95), suggesting that antibodies that disrupt rosetting may confer protection. The same study found that IgG to SM-associated CIDRγ12 was also significantly higher in UM compared to SM. Another study found that FcγRIIIb binding antibodies to CIDRγ12 were significantly higher in UM than CM and this was one of seven key features predictive of protection against CM (102). Though there is limited direct evidence linking the CIDRγ12 to rosetting, other CIDRγ domains have been linked to rosetting. Evidence suggests that CIDRγ2 can mediate rosetting. One study showed that the CIDRγ2 domain of 3D7A parasites binds glycophorin B on uninfected erythrocytes to mediate robust, plasma-independent rosetting, and anti-CIDRγ2 antibodies specifically inhibited rosetting in 3D7A parasites in a dose-dependent manner (9). Interestingly, anti-CIDRγ2 antibodies did not affect rosetting of another parasite line FCR3, whose rosetting involves IgM and α2-macroglobulin. In the FCR3 parasite, antibodies to DBL1α-CIDRβ blocked rosetting (103), highlighting that there are multiple, domain-specific mechanisms of rosette formation. These findings suggest a possible link between rosetting and the pathogenesis of CM, highlighting the need for further investigation into the underlying mechanisms and the potential for antibodies targeting rosetting domains to offer protection against CM.

Some rosetting parasites bind IgM and IgM facilitates the adhesion of the IEs to uninfected erythrocytes (104). IgM-positive rosetting parasites express distinct PfEMP1 variants with specific N-terminal and DBL domains, and polyclonal IgG antibodies raised against these variants could inhibit rosetting across different strains (73), indicating strain-transcending potential. In contrast, IgM-negative rosetting parasites exhibited strain-specific antibody activity (73). These strain-transcending antibodies represent the kind of broader protection that would be ideal for developing a preventative measure against CM. However, there is no data on which rosetting strains are expressed in CM or whether antibodies toward them provide protective immunity. These aspects require further investigation.

### 3.4 Functional features of antibodies protective against cerebral malaria

PfEMP1-specific antibody responses may offer protection against malaria by eliciting multiple effector functions, including blocking IE adhesion (73, 105) and promoting immune clearance via ADCP (76, 101, 105) and antibody-dependent cellular cytotoxicity (ADCC) (106–108).

Complement enhances antibody-mediated inhibition across multiple parasite stages, including sporozoites (CSP), merozoites (MSP1), and sexual forms (Pfs230) (104, 109), however the role of complement fixing antibodies against PfEMP1 and IEs is less clear (110). Recent findings emphasize the role of antibody-dependent effector functions in distinguishing CM from UM, identifying seven key antibody features predictive of malaria severity (102). Among these were antibodies that fix C1q (targeting the ICAM1 binding IT4VAR13_DBLβ) indicating that complement-mediated effector functions may be a protective mechanism. While these insights offer a valuable starting point, it is important to note that findings based on recombinant proteins may not fully capture native PfEMP1 conformation or function *in vivo*. Given that IgG1 and IgG3 dominate the PfEMP1-specific response in semi-immune individuals (111–113) and are potent complement activators, their ability to fix C1q may contribute to protection against CM. However, a study found that complement-mediated opsonization of IEs is limited in efficacy, potentially due to the patchy distribution of PfEMP1 on the IE surface (111), which could limit IgG hexamerization—an essential step for effective C1q activation (114). Although antibodies that fix C1q have been suggested to play a protective role in CM, it is important to confirm if complement deposition does occur on the IE and whether it enhances complement-mediated phagocytosis. Future studies should elucidate how C1q-fixing antibodies targeting PfEMP1 contribute to protection against CM, for better prognostic and therapeutic strategies.

Fc mediated functions may protect against CM; antibody features associated with protection from CM include FcγRIIIb-binding antibodies targeting ICAM1 binding DBLβ3 PfEMP1 domains (112). In the same study, Malawian children with UM had higher neutrophil-mediated phagocytosis of IEs expressing ICAM1 and EPCR-binding PfEMP1 (3D7VAR04) than children with CM (112), suggesting a protective role of FcγRIIIb-dependent phagocytosis. In contrast, children with CM had higher neutrophil-mediated phagocytosis of ICAM1 and CD36-binding IEs (IT4VAR13), indicating a difference in immune targeting of variant surface antigens. FcγRIIa can work synergistically with FcγRIIIb to enhance antibody-dependent neutrophil phagocytosis of merozoites (115) and sporozoites (116), it is currently unknown whether this is similar for PfEMP1. This suggests that exploring Fc-mediated immune functions, such as complement activation, ADCC, and FcγR engagement, could provide stronger predictive value for clinical outcomes than total neutralizing antibodies alone.

### 3.5 Fucose-free PfEMP1-specific antibodies

Antibody-dependent immune effector functions depend on IgG binding to FcγR on immune cells, a process influenced by glycosylation (the presence of sugar molecules on the antibody’s Fc region), which can significantly influence downstream immune functions. One key glycosylation modification is Fc-afucosylation, the absence of fucose on the biantennary glycan at asparagine 297 (N297) in the IgG Fc region. This enhances IgG binding to FcγRIIIa, greatly amplifying ADCC activity (117). Afucosylated IgG antibodies targeting enveloped viruses or alloantigens are highly effective at ADCC, as they can bind the FcγRIIIa receptor on immune cells up to 20 times more strongly (117–119).

A study on IgG afucosylation in pregnancy-associated malaria highlighted the potential importance of afucosylated antibodies in malaria immunity (107). Researchers found that naturally acquired IgG targeting pregnancy-associated PfEMP1 (VAR2CSA) was afucosylated and remained stable over time, whereas vaccination did not induce afucosylated antibodies (107). Notably, only afucosylated VAR2CSA-specific IgG could induce natural killer (NK) cell degranulation (107). In a more recent study, children with Fc-afucosylated IgG1 targeting the PfEMP1 variant HB3VAR06 were less likely to be anemic. Fc-afucosylation of these antibodies is acquired through repeated malaria exposure and persists over time. Protection from anemia was associated with immune cell activation via FcγRIIIa, rather than complement-mediated lysis, likely due to the uneven distribution of PfEMP1 on IEs (120), suggesting that Fc-afucosylation enhances antibody function by improving IgG binding to FcγRIIIa, which boosts NK cell activation and IE clearance. However, the role of NK cells in CM may be a double-edged sword, as children with CM have more activated NK cells than those with SM or UM (121).

Despite growing interest in antibody glycosylation, there is limited data on the role of afucosylated IgG antibodies in CM.

## 4 Where can we go from here?

Current evidence suggests that antibodies targeting PfEMP1 have the potential to protect against CM, but further research is needed to determine which PfEMP1 targets are most important for immunity. Longitudinal studies indicate that hyperimmune individuals in endemic areas develop antibodies against EPCR-binding CIDRα1 domains, which may limit clinically dense malaria (84, 86). Both EPCR-binding domains and those with dual binding to EPCR and ICAM1 have been associated with CM (38–40). It is likely that the determinants of protective immunity extend beyond the neutralizing capacity of PfEMP1-specific antibodies and understanding how adhesion inhibition and immune effector functions cooperate to eliminate infections will be important.

Our understanding of naturally acquired immunity to malaria, and CM in particular, remains incomplete. One key gap is our limited understanding of why some PfEMP1 variants trigger strong, protective antibody responses while others evade immunity, leaving young children—despite early exposure—more vulnerable to CM compared to older children and adults who develop sustained responses against EPCR-binding (86) and ICAM1-binding (79) domains. When considering a determinant of antibody function, such as afucosylation—acquired over repeated exposure and persisting over time—we recognize its potential to significantly enhance immune effector functions like ADCC. It would be interesting to explore how afucosylation evolves over time, whether it contributes to age-related differences in immunity, and how relevant it is for protection (**Table 1**). The gold standard for addressing some of these questions would be longitudinal cohort studies in malaria-endemic regions that follow individuals from infancy to adulthood. Such studies would provide comprehensive data on how antibody responses to PfEMP1 develop, persist, and change over time. However, realistically, these studies may not be feasible due to several limitations, including their high cost, the substantial manpower required for long-term follow-up, and the logistical challenges associated with maintaining consistent data collection over many years.

**Table 1:** Research gaps and proposed approaches.

Knowledge gap	Proposed approach
1. The roles of non-binding PfEMP1 domains (e.g. DBLα, CIDRγ2, γ6, γ12) in modulating sequestration and rosetting, and their potential as protective antibody targets.	Assess how non-binding domains in PfEMP1 variants associated with CM affect binding affinity and test the functional efficacy of domain-specific antibodies in blocking adhesion and protecting against CM.
2. Role of Fc glycan (e.g. afucosylation) and other post-translational modifications in antibody effector function.	Combine mass-spectrometry mapping of post-translational modifications on anti-PfEMP1 IgG from CM vs UM patients with functional assays (ADCC, ADNP).
3. Limitations of recombinant-domain multiplex assays vs native-IE assays.	Parallel systems serology profiling: compare recombinant-domain enzyme-linked immunosorbent assays/microarrays with whole-IE binding/phagocytosis/complement-fixation assays to validate correlates.
4. Techniques to minimize antigen switching	Develop and maintain antigenically stable parasite lines (single-var expressors)
5. Need for robust binding-inhibition assays that reflect *in vivo* conditions.	Employ 3D microvessel flow models and endothelial organoids to test antibody or monoclonal antibody blockade of IE sequestration under physiological shear stress.

Post-translational modifications do not only occur on antibodies but can also occur on FcγRs located on immune effector cells and regulate antibody effector function. For example, ligand−induced ubiquitination of the FcγRIIIa ζ−chain targets the receptor for degradation, reducing its surface expression and dampening natural killer cell activation (122). In the context of anti−PfEMP1 antibodies, investigations into antibody Fc and FcγR modifications beyond glycosylation remain scarce. Mass spectrometry–based mapping of post-translational modifications (123) in anti-PfEMP1 antibodies from CM patients and in-silico structure-based predictions of potential modification sites (124) offer powerful strategies for identifying which post-translational modifications are important in CM.

EPCR-binding and dual-binding PfEMP1 variants are closely associated with CM, leading to the natural hypothesis that antibodies targeting these variants would provide protection. However, the data thus far on both DC8- and DC13-containing PfEMP1 variants, while suggestive of protection, is not conclusive. This presents a gap that needs further exploration: which isolates of these variants are most immunogenic, which peptides within them are immunogenic, and whether these responses are protective. Beyond antibodies to CIDRα1 and DBLβ domains, other regions of PfEMP1—such as DBLα and CIDRγ2 domains involved in rosetting, and CIDRγ6 and CIDRγ12 domains with unknown binding phenotypes—may also influence PfEMP1-mediated adhesion. It is imperative to assess how their presence in EPCR-binding PfEMP1 variants enhance CIDRα1’s interaction with EPCR and evaluate the protective potential of antibodies against each of these domains in CM.

Monoclonal antibodies like C7 and C74, which target the CIDRα1 domain of PfEMP1, show promise in preventing IE sequestration in CM by blocking adhesion to endothelial cells (125). Identifying post-translational modifications that are important for protection could help enhance the blocking capabilities of broadly inhibitory monoclonal antibodies, such as these.

How broad must the antibody repertoire be to confer effective protection, and what is the best way to measure immunity? Studies like Walker et al. (102) demonstrate the power of a systems serology approach in identifying targets and features of a possible protective antibody response. The study also illustrates potential shortcomings of recombinant protein-based multiplex immunoassays, which did not correlate well with assays using whole IEs (102). This discrepancy may be due to differences in antigen presentation and Fc receptor engagement, underscoring that assays based on individual recombinant PfEMP1 domains may not fully capture immunity to PfEMP1 in its native configuration or full-length proteins (126). PfEMP1 antigenic switching (127) remains a significant challenge for *in vitro* studies, and it will be critical to obtain antigenically stable parasite lines that accurately reflect the antigenic diversity in natural infections.

While this review has focused on neutrophils primarily in terms of antibody-mediated phagocytosis, the work by Zelter et al. (128) demonstrates that neutrophils can also directly recognize directly recognize and kill infected erythrocytes via ICAM-1, independently of antibodies. This suggests that, in addition to their antibody-dependent phagocytic function, neutrophils may exert selective pressure on the parasite population: IEs expressing ICAM1-binding PfEMP1 are preferentially eliminated, forcing surviving parasites to downregulate or switch away from these variants. This “filtering” mechanism may contribute to protection and raises an important question for protective immunity: are antibody-mediated opsonic phagocytosis and ICAM1–mediated killing both necessary for effective protection against severe disease?

Another gap is the lack of robust binding inhibition assays, and how these interactions influence immune effector functions remains largely unknown. Most binding assays to date have relied on 2D systems, such as flat layers of cultured cells or immobilized receptors, where parasites adhere under static conditions (129, 130). Recent work using engineered 3D human brain microvessels has shown IEs cytoadhere via PfEMP1–endothelial receptor interactions, mature, and rupture within a physiologically relevant microvascular environment (70, 131, 132). This binding triggers endothelial activation, including upregulation of ICAM1 and inflammatory signaling, promotes focal barrier disruptions and endothelial apoptosis, and elicits a stress response profile distinct from that differ from conventional 2D monolayers (132, 133). These 3D microvessel models are already proving valuable in investigating PfEMP1-mediated adhesion and offer a versatile platform for future research to assess how changes in the microenvironment influence PfEMP1 expression, transcription, and binding, as well as to evaluate the efficacy of broadly inhibitory monoclonal antibodies, both current and in development.

In conclusion, there is still much to learn about the antibody responses to PfEMP1 that provide protection from CM. Current research has laid a strong foundation for asking the crucial questions that will guide us toward those answers. There are many exciting future directions, including exploring monoclonal antibodies, post-translational modifications, and 3D vascular models, which may bring us closer to a deeper understanding of the immune mechanisms at play and potentially effective interventions.
